# Global suppression of electrocortical activity in unilateral perinatal thalamic stroke

**DOI:** 10.1111/dmcn.12365

**Published:** 2014-01-11

**Authors:** Liudmila Kharoshankaya, Peter M Filan, Conor O Bogue, Deirdre M Murray, Geraldine B Boylan

**Affiliations:** 1 Department of Paediatrics and Child Health, University College CorkCork, Ireland; 2 Neonatal Brain Research Group, Irish Centre for Fetal and Neonatal Translational Research (INFANT), Cork University Maternity HospitalCork, Ireland; 3 Department of Neonatology, Cork University Maternity HospitalCork, Ireland; 4 Department of Radiology, Cork University HospitalCork, Ireland

## Abstract

We present an unusual case of persistent generalized electroencephalography (EEG) suppression and right-sided clonic seizures in a male infant born at 40^+2^ weeks' gestation, birthweight 3240g, with an isolated unilateral thalamic stroke. The EEG at 13 hours after birth showed a generalized very low amplitude background pattern, which progressed to frequent electrographic seizures over the left hemisphere. The interictal background EEG pattern remained grossly abnormal over the next 48 hours, showing very low background amplitudes (<10*μ*V). Magnetic resonance imaging revealed an isolated acute left-sided thalamic infarction. This is the first description of severe global EEG suppression caused by an isolated unilateral thalamic stroke and supports the role of the thalamus as the control centre for cortical electrical activity.

The incidence of perinatal arterial stroke is estimated to be one in 2300 to 4000 live births,^1^ and over 80% of cases involve the middle cerebral artery or its branches.^2^ Perinatal thalamic stroke is uncommon, but the true incidence is unknown. Most arterial blood supply to the thalamus originates from the posterior cerebral artery.^3^ The thalamus is critical to brain function: thalamic nuclei connect multimodal motor and sensory neurons between the cortex and subcortical structures and play a role in cognitive function, mood, and awareness.^4^

Generalized electrocortical suppression in term newborn infants is characteristic of widespread cortical injury such as severe hypoxic–ischemic encephalopathy, bilateral cortical infarction/haemorrhage, inborn errors of metabolism, or cortical dysgenesis.^5^ Radiological and biochemical investigations ruled out these differential diagnoses in our case, whereas magnetic resonance imaging (MRI) revealed an isolated acute ischemic lesion in the left thalamus. Written parental informed consent was obtained for publication of this case report.

## Case Report

A 20-year-old Irish Caucasian woman, gravida 2, para 1, with a history of hypothyroidism and a body mass index of 44 delivered a male infant (birthweight 3240g) at 40^+2^ weeks' gestation. During the pregnancy she had a 1-week viral illness at 9 weeks' gestation, suspected rupture of membranes at 29 weeks, and a non-substantial vaginal bleed at 37 weeks. After spontaneous onset, labour was augmented with oxytocin infusion. The duration of the first stage of labour was 6 hours and the second stage was 54 minutes. Cardiotocography was reactive throughout labour. After a vaginal cephalic delivery, meconium grade II was present but cord pH was not recorded as the infant was in a good condition with Apgar scores of nine at 1 and 5 minutes. The infant received routine postnatal care, but was noted to have persistent irritability during the first 12 hours after birth, which was attributed to a caput succedaneum. At 13 hours of age he developed rhythmic jerking of the right arm and leg, associated with mouthing and cyanosis and was immediately transferred to the neonatal intensive care unit. Initial management included intravenous phenobarbitone (20mg/kg loading dose) and antibiotics, and multi-channel video electroencephalography (EEG) monitoring started soon after. Investigations including full blood count, electrolytes, blood glucose level, coagulation screen, and C-reactive protein were all normal, and blood cultures were negative. Seizures re-appeared at 18 hours and two further doses of phenobarbitone (10mg/kg) and phenytoin (20mg/kg) were administered to achieve clinical seizure control at 31 hours. Maintenance phenytoin (5mg/kg daily) was administered for the subsequent 4 days. On examination, during seizure-free periods, the infant showed reduced activity with moderate axial hypotonia and mild peripheral hypertonia.

**Figure 1:**
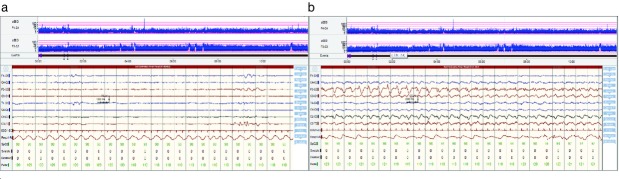
Dual-channel amplitude-integrated electroencepaholgrapy (EEG) and EEG in a male term infant with left-sided thalamic stroke. (a) The background EEG from approximately 14 hours after delivery showed severe global suppression with a low-voltage burst suppression pattern (<10*μ*V). No state cycling was evident. This continued for up to 48 hours, after which a gradual increase in voltage was seen to approximately 25*μ*V. (b) Electrographic seizures show a clear focus over the left hemisphere with some right sided spread.

Magnetic resonance imaging was performed on day two using a 1.5T MRI (Siemens AG, Erlangan, Germany). Diffusion-weighted imaging (TR 800, TE 106, EPI 128, Av 3, B = 1000, voxel size 1.8mm × 1.8mm × 4.0mm) demonstrated restricted diffusion in the left thalamus and left internal capsule, in keeping with acute ischemia. There was no evidence of internal cerebral venous thrombosis. The basal ganglia, right thalamus, right internal capsule, and both cerebral hemispheres were normal in appearance (Fig.1).

A neurological examination on day three, including an Amiel-Tison assessment, revealed marked axial hypotonia and poor primary reflexes. Low-dose computed tomography of the skull, performed because of a concern about the infant's head shape, confirmed bilateral coronal suture synostosis. Extensive metabolic screen and karyotype test results appeared normal. Serum levels of antiepileptic drugs were also normal.

Formula feeds were established on day three. The infant was discharged home on day nine, feeding orally, but with significant head lag. A hearing screening (otoacoustic emission) on discharge demonstrated clear response from the right and left ears. At a 2-month clinic follow-up, head lag was mild with reduced movements of the right upper limb.

At 9 months of age, the infant was assessed using the Griffiths Scales of Infant Development by an independent paediatrician (DMM). The infant was rolling over, and sitting with support. Tone, power, and reflexes appeared normal in all four limbs, with no evidence of cerebral palsy at this early stage. Visual fixation was reduced and the infant displayed poor oculomotor control. The gross motor subscale on the Griffiths Scales was 80, and reduced scores were seen across all subscales with a total developmental quotient of 62 (<2 SD below the mean).

**What this paper adds**

- In neonates global electrocortical suppression may occur due to a focal/unilateral lesion.
- Unilateral perinatal thalamic infarction can present with global electrocortical suppression and seizures.

## EEG findings

A continuous 8-channel video-electroencephalogram (Nicolet One ICU Monitor, Neuro-Care, Carefusion, Middleton, WI, USA) with dual-channel amplitude-integrated EEG was started on admission to the neonatal intensive care unit at 14 hours after birth. Cerebral activity using nine scalp electrodes (F3, F4, C3, C4, T3, T4, O1, O2, Cz), electrocardiography, and respiration movements were recorded simultaneously. An experienced neonatal neurophysiologist (GBB) performed visual EEG analysis.

**Figure 2:**
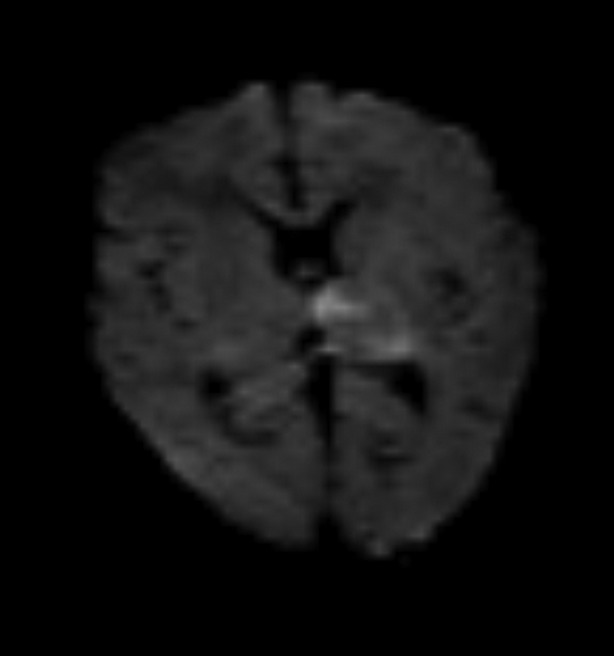
Magnetic resonance imaging on day two. Axial diffusion-weighted imaging demonstrating restricted diffusion in the left thalamus and left internal capsule.

The initial EEG showed almost complete global suppression of electrocortical activity, with amplitudes of less than 10*μ*V with no electrographic seizure activity and no evidence of sleep–wake cycling (Fig.2a).

Electroclinical seizures were recorded at 18 hours after birth, demonstrating focal left-sided biphasic spike-wave discharges with some secondary generalization (Fig.2b).

Clinical seizure control was achieved at 31 hours, whereas electrographic seizures continued until 40 hours. Overall, the infant had a total of 106 minutes of electrographic seizures over a period of 42.5 hours (36 min with clinical correlates). The background EEG remained suppressed for up to 48 hours after birth, after which a gradual increase in voltage was seen to approximately 25*μ*V. By day 5, the EEG amplitude had recovered to approximately 50*μ*V, with disrupted sleep–wake cycling.

## Discussion

To our knowledge, this is the first report of global EEG voltage suppression associated with unilateral thalamic infarction in a neonate. The clinical presentation suggested a unilateral neonatal stroke involving the middle cerebral artery. Typical EEG characteristics of middle cerebral artery stroke include voltage reduction or sharp waves localized to the region of injury and intermittent seizures.^6^ In many cases, sleep cycling is preserved. In unilateral perinatal stroke, the EEG may show asymmetry and asynchrony between hemispheres^7^; however, generalized EEG suppression has not previously been described.

This was a very different EEG pattern and atypical for perinatal stroke. Most worrying was the length of time that the EEG remained generally suppressed, i.e. 4 to 5 days. In term infants, the early postnatal EEG should show moderate voltage continuous mixed frequency activity with clear sleep cycling.^8^ Sleep cycles emerged in this infant but were disrupted. In term newborn infants with hypoxic–ischemic encephalopathy, persistent EEG suppression at 48 hours and an absent sleep–wake cycle are poor prognostic signs.^9^

The thalamus has a critical role in global brain function. Previous reports, from children and adults with unilateral thalamic infarction, have described bilateral generalized spike-wave discharges^10^,^11^ and bilateral reduction of the number of sleep spindles.^12^ Contralateral cortico-cortical projection of excitation from the site of injury across the corpus callosum has been suggested as a possible explanation.^10^ MRI mapping of the adult human brain has demonstrated possible direct connectivity from lateral and medial thalamic nuclei to the contralateral subcortex through the corpus callosum.^13^ Similar direct contralateral thalamo-cortical projections have been reported to be a common feature of the developing brain in several mammalian species with a large amount of transitional cortex.^14^ In the human brain, the transitional cortex (cortical subplate) disappears at 6 months after birth but plays a key role in the formation of thalamo-cortical and cortico-cortical connectivity. Thalamo-cortical and cortico-cortical axons form transitional synapses in the subplate before they grow and relocate to the cortical plate. These transitional synapses constitute fetal circuitry. The subplate and fetal circuitry usually disappear between 35 and 37 weeks of gestation but can remain longer past term age.^15^ We hypothesize that transitional fetal thalamo-corticocortical circuitry in a term infant may significantly contribute to direct contralateral thalamo-cortical suppression during the acute phase of unilateral thalamic injury.

The amount and distribution of the remaining subplate in the neonatal period may differ individually, and this is not the only factor affecting the EEG background. For example, bilateral discontinuity and left-sided central sharp waves were reported in a newborn infant with bilateral paramedian thalamic and mesencephalic infarcts.^16^ The recording, however, was performed shortly after the phenobarbital load, and an additional MRI finding in this case was restricted diffusion in the right periatrial white matter.

Although the most common cause of persistent generalized EEG suppression would be a devastating global hypoxic–ischemic injury, this did not fit with the clinical picture in this case: the infant had normal cardiotocogram and Apgar scores at birth, and was clinically well until seizures manifested at 13 hours of age. MRI diffusion-weighted imaging on day two did not reveal any cortical injury.

## Conclusion

We have described an alternative cause for severe generalized voltage suppression in the EEG of a term infant in the early postnatal period. Perinatal thalamic infarction should be considered in the differential diagnosis in term infants who present with seizures when previously well.
