# Supplementary material for: Global suppression of electrocortical activity in unilateral perinatal thalamic stroke
Source: Dev Med Child Neurol. 2014 Jan 11;56(7):695–8. doi: 10.1111/dmcn.12365 (PMC4190684; doi:10.1111/dmcn.12365)
Supplement: Related texts

# Correction: Erratum

**Published:** 2014-01-11

**Infantile neuroaxonal dystrophy caused by uniparental disomy.**^1^

In the above article, the name of the fourth author should have been given as Manju A Kurian. We apologize for the error.

## References

[b17] Solomons J, Ridgway O, Hardy C (2014). Infantile neuroaxonal dystrophy caused by uniparental disomy.. Dev Med Child Neurol.
